# The role of digital and algorithm-based alert systems in policing mental health crises: a scoping review

**DOI:** 10.1186/s40352-025-00385-x

**Published:** 2025-12-02

**Authors:** Therese Bäckman, Elin Sandegård, Charlotta Thodelius

**Affiliations:** 1 Faculty of Police Work, University of Borås, Borås, Sweden; 2 Department of Law, University of Gothenburg, Gothenburg, Sweden

**Keywords:** Mental illness, Policing, Digital alert system, Information sharing

## Abstract

**Background:**

This scoping review explores how police and health care use digital and algorithm-based alert systems to flag individuals with mental illness. Eight studies were included, covering both police systems and health care-initiated systems to alert law enforcement.

**Result:**

The findings suggest that digital and algorithm-based alert systems can support decision-making and improve police interactions with individuals experiencing mental health issues. However, in health care settings the systems are motivated by workplace safety concerns and in policing from an efficiency perspective, stressing collaborative crisis response between police and health care services. Additionally, information sharing and collaboration between police and health care services benefit from such technology. However, the use of these systems also raises ethical concerns and risks of potential stigmatization.

**Conclusion:**

The review highlights the importance of balancing efficiency and ethical consideration when implementing digital and algorithm-based alert systems in mental-health related policing. Thereto forthcoming studies need to consider if and how frontline policing will benefit from digital and algorithm-based alert systems.

## Background

Police officers use of digital- and algorithm-based alert systems to identify individuals with mental health issues is a potentially important but understudied practice. This explorative scoping review seeks to map contemporary research and contribute to the emerging discussion on implementing digital- and algorithm-based alert system.

Police officers frequently respond to mental health crises, and without adequate training or mental preparedness, there is an increased risk for escalation and use of force in these situations (Wittmann, Jörns-Prestanti & Groen, 2021; Watson et al., 2019). Mental preparedness can be defined as a psychological state of being ready to handle challenges by remaining calm and making informed decisions during high stress situations (cf. Andersen et al., 2015; Newton & Smith, 2024).

The question of mental health crisis response training has gained increased attention, but there is also a great variation between departments and countries in training description, time in training and use of response models (Hails & Borum, 2003; Wood & Watson, 2017; Kane et al., 2017). In previous research a variation of response models has been seen as promising, such as crises interventions teams (CIT), Mental Health first aid training and co-response teams (Arazan & Weich, 2024; Crisanti et al., 2022; Chungheyon, Bitna & Kruis, 2021; Lowder et al., 2024; Seo, Kim & Kruis, 2021). The general aim with the models is to enable an effective and safe intervention, minimizing the risk of escalating the situation and the use of force (Gill, Jensen & Cave 2018). Use of force in policing refers to the application of physical power by law enforcement to maintain order, ranging from verbal to lethal force depending on the situation. However, use of force needs to be objectively reasonable given the circumstances. According, to Andersen et al. (2015) training and fostering resilience in policing can be a way to handle questionable use of force actions.

In addition, previous research stresses the importance of sharing information between police and health care staff and collaboration in these encounters to enable both mental preparedness, proper decisions on intervention and respectful conduct in policing mental health crises (cf. Schulenberg, 2016; Seo, Kim & Kruis, 2021).

Current advances in technology have led to development of digital or algorithm-based alerts systems in policing (Langton et al., 2021), but little is known of use, usability and extent in policing mental health crisis. Systems called digital alert systems, digital flags, or use of vulnerable person databases or screeners, can be potentially helpful for the police in interactions with individuals with mental health conditions. The system helps with early identification and intervention, by preparing the police for potential challenges, the need to take any special consideration and prevent negative interactions (Compton et al., 2017; Hartford et al., 2005; Kane et al., 2018). Some of the databases also facilitate internal communication by attaching electronic caution flags to individuals’ names, using algorithms. These algorithms in combination with address and key words categorizes individuals into definite, probable, and possible person with mental illness (Hartford et al., 2005; Kane et al., 2018). The technology development has also led to a development of algorithm-based screeners, which can be used in situations when the police encounter individuals with suspected mental health crises (cf. Gooding, 2019). Similar systems have also been developed in the health care sector, these health care-initiated systems can be used to alert police, security guards and/or staff in cases with an increased risk for violent encounters with patients (Bach et al., 2018).

The development of digital and algorithm-based alert systems raises new ethical and juridical issues in policing, which is of importance to address. Especially, how to find the balance between the need of enabling early identification of individuals to improve the outcome of mental health-related encounters and the use of sensitive information, personal data and risk of stigmatization (Aden, 2020; Langton et al., 2021; Wood, Watson & Barber, 2021).

This scoping review aims to explore and synthesize current knowledge about the implementation and use of digital and algorithm-based alert systems in policing, in relation to mental health crises. The review also includes health care-initiated systems used to alert the police. By comparing these practices, the review seeks to identify motivations, practical applications, and the benefits and drawbacks of these systems.

We will address the following three research questions: (1) What are the primary motivations underlying the implementation of digital and algorithm-based alert systems within the police organizations on one hand, and within health care services on the other? (2) In what way are the different systems used? (3) What advantages and disadvantages have been identified in relation to these alert systems, particularly regarding effectiveness, legality and ethical concerns.

By summarizing the current knowledge in the field of digital and algorithm-based alert systems of relevance for policing, we can contribute to the discussion of implementing and use of the system.

## Method

This scoping review seeks to map contemporary research on digital- and algorithm-based alert systems, and the choice of method relies on Chambers et al.’s (2011) notion that the scoping review method is suitable for exploring the current knowledge, especially in an inter- and transdisciplinary field. Particularly, since the method allows an integration of perspectives and methodological approaches by expansive inclusion criteria practice within the method (Munn et al., 2018). The method also adheres to strict methodological standards to map out the nature of the research field (Arksey & O’ Malley, 2005).

To ensure a systematic search process, we chose to construct our search string in relation to the PICo-model (Munn et al., 2018). PICo, stands for population, phenomena of interest and context, and each block is a part in the search string, to identify articles which intertwine both population, phenomena and context (Table 1). As seen in Table 1, our population was defined as police or the practice of policing, namely how the police perceive the use of algorithm based alert systems. The phenomena of interest are different expressions related to the use algorithm based alert systems narrowing down the context to mental, psychological, behavioral health and emotional well-being.

**Table 1:** PICo for the applied search string

P = Population	Policing OR Police
I = Phenomena of interest	Digital flagging OR digital alert* OR electronic flagging OR automated alert* OR vulnerable persons database OR early warning system
Co = Context	Mental health OR emotional well-being OR psychological health OR behavioral health

For the database searches the different blocks related to the booel AND, creating the following search string: (“Policing” OR “Police”) AND (“digital flagging” OR “digital alert*” OR “electronic flagging” OR “automated alert*” OR “vulnerable persons database” OR “early warning system”) AND (“mental health” OR “emotional well-being” OR “psychological health” OR “behavioral health”).

**Fig. 1:**
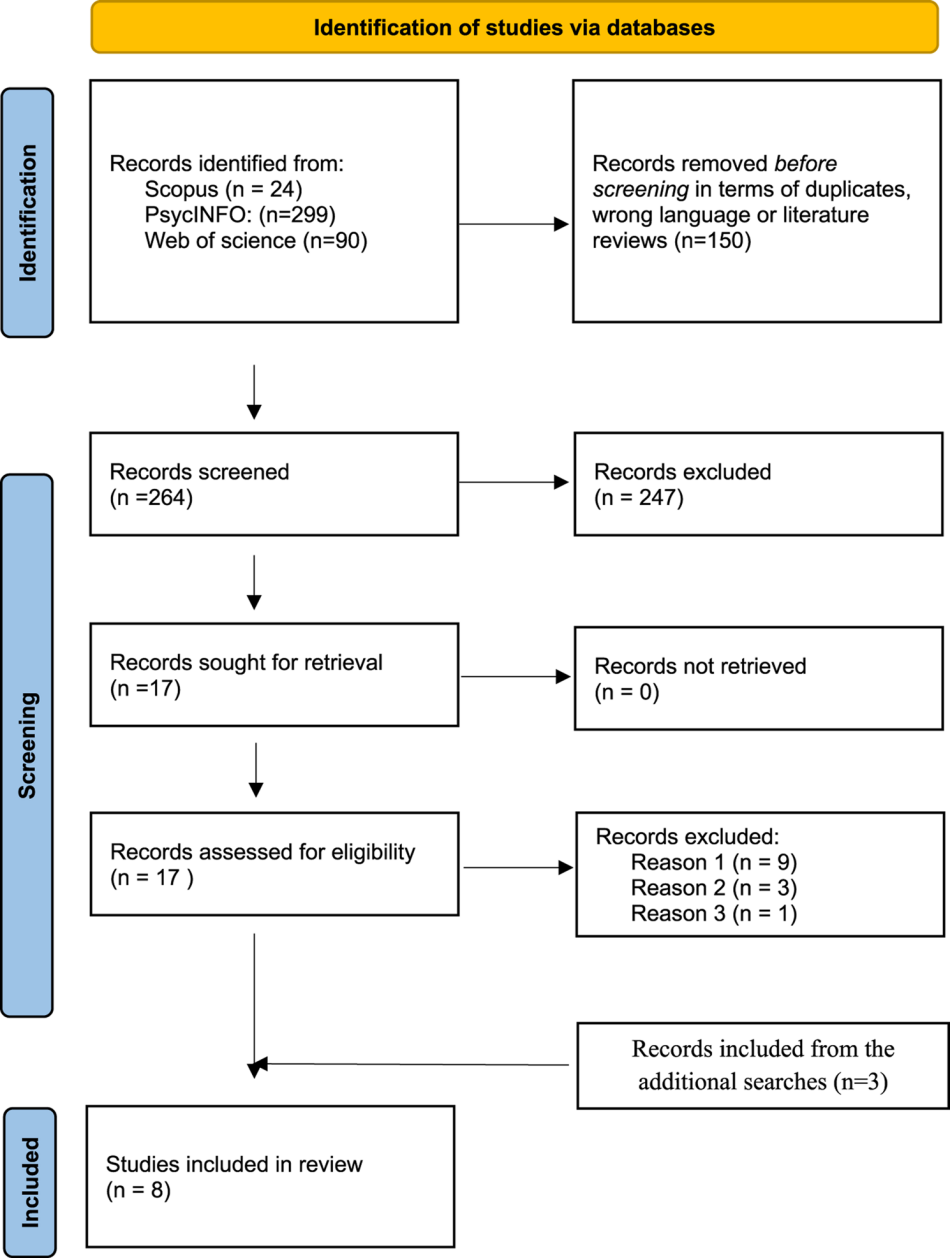
Process of selection of articles included in the scoping review

The search process, extraction of data and analysis was informed by the Preferred Reporting Items for Systematic Reviews and Meta-Analyses (PRISMA) approach to systematic reviews (see Fig. 1) and a scooping review protocol (Moher et al., 2009; Hand, Mitchell & DeGregory, 2016).

The scoping review consisted of two searches, one initial search and one additional search, the database searches were conducted by two of the authors (TB and CT). The screening process was conducted by all three authors.

All studies retrieved were screened in two steps: first by title and abstract, and subsequently by full text. The screening process followed the inclusion and exclusion criteria defined in Table 2. To ensure that the included studies focused on the current state of the art regarding digital and algorithm-based alert systems in policing, in relation to mental health crises, we excluded all articles published before 2010. We also verified that the included studies were peer-reviewed to ensure research quality. Therefore, we only included empirical studies to highlight practical use of digital- and algorithm-based alert systems.

**Table 2:** Inclusion and exclusion criteria

Inclusion	Exclusion
Published between 2010–2024	Published before 2010
Peer reviewed research articles	Literature reviews
	Conference proceedings
	Grey literature
Open Access and available full text	-
Written on English	All other languages
Use of intervention: empirical studies	Literature reviews
	Theoretical studies

### Initial search

The databases chosen for the initial search were Scopus, PsycINFO and Web of Science, due to the subject’s interdisciplinary characteristic (policing, health care, law, digitalization and mental health). As shown in Fig. 1, the initial search identified 413 records, of which 150 were excluded prior to screening. The reason for exclusion in this step was mainly the occurrence of duplicates, literature reviews and articles written in languages other than English. Initially, titles and abstracts were screened, leading to the exclusion of and 247 records that did not meet the inclusion criteria based on title and/or abstract content. 17 articles were sought for retrieval and read in full text by all three authors. Of these, nine were excluded due to wrong scope (reason 1), three due to wrong population (reason 2) and one due to wrong context (reason 3). The initial search and screening process resulted in the inclusion of four studies in the review.

### Additional searches

Given the limited number of studies included from the initial two searches, two additional searches were conducted. The first additional search was conducted in the Criminal Justice database and the second was hand-picking of articles. Due to the database structure, the search string differed a bit from the initial search but kept our core terms. The records identified in the first additional search (*n* = 80), were screened by the same inclusion and exclusion criteria as in the initial search. After the first initial exclusion of the Criminal Justice database, 22 records were assessed by abstract screening, and two records were sought for retrieval. Of these, one study was included in the review. The second additional search conducted by a hand-picking of articles, resulted in 39 records. After the initial screening, 14 records were sought for retrieval and two studies were included in the review.

### Data extraction and analysis

Data was extracted from each of the eight articles and recorded in an Excel file. The extracted data included authors, year of publication, country where the study was conducted, method (qualitative or quantitative) and type of digital- or algorithm-based alert system (e.g. systems for the police or health care-initiated system).

A thematic analysis was conducted to identify themes and sub-themes related to the studies research questions. This involved an iterative process with reading and re-reading the included studies (cf. Braun & Clark, 2006; Nowell et al., 2017). The analysis was conducted collectively by all three authors. The process involved familiarization with the material by reading the studies thoroughly, generating initial codes, searching for themes and reviewing them before defining the final themes. This approach allowed for a structured and in-depth examination of recurring concepts and key insights across the selected studies. The following three themes were identified: (1) motives and needs; (2) organizational factors and (3) dilemmas and ethical issues.

## Results

The review included eight studies divided into two categories: digital and algorithm-based alert systems for the police (*n* = 5) and health care-initiated systems (*n* = 3). Digital alert system is based on previous information regarding the individual (information sharing through electronic health records). Algorithm-based alert system consists of algorithmic risk prediction and assessment of the situation (screeners). Of the alert system used by the police, four studies investigated the use of screeners in policing.

**Fig. 2:**
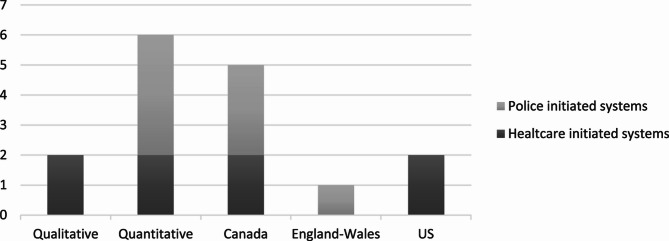
Distribution between included articles regarding method and countries

As seen in Fig. 2, six of eight studies apply a quantitative method, and most of the studies were conducted in Canada (*n* = 5), followed by US (*n* = 2) and England-Wales (*n* = 1). The studies were mainly published in journals focusing on psychiatric or mental health (*n* = 4), followed by health care-oriented journals (*n* = 2) and one (*n* = 1) in journals focusing on threat assessment or policing (see Appendix 1).

### Motives for and needs of digital and algorithm-based systems

The use of digital or algorithm-based alert systems are described as an intervention in health care settings to reduce the risk of workplace violence, especially in emergency departments (Haimovich et al., 2024; Paterson et al., 2019: Weinberger et al., 2018). The systems seem to enable internal and external communication in risk situations and directly facilitate patient and staff safety (Paterson et al., 2019). In contrast to health care initiatives, systems for the police were motivated by various reasons, such as information sharing between authorities (Hirdes et al., 2020; Hoffman et al., 2016), cost effectiveness and justification of budget for non-crime events (Hoffman et al., 2021), assessing risks, improving police response, facilitating interaction and avoiding violent encounters (Kane et al., 2018; Sanders & Lavoie, 2020).

A re-occurring theme in all studies, regardless of system, is the need to enable communication, collaboration and information sharing, both within and between the organizations. An identified problem in collaboration and communication between police officers and health care professionals seems to be a gap between the different missions which hinders communication. Traditionally, the police have focus on public safety and health care professional on diagnosis and care in meetings with individuals with suspected mental illness. Sharing information through joint screening systems can foster increased consensus and a better understanding of the differing mandates of involved organizations (Hoffman et al., 2016).

Another aspect emphasized is the possibility of psychological readiness for the police in encounters with individuals in mental health crisis. In the studies which included screeners for assessment in an encounter, the police often used previous knowledge about the person to prepare (Sanders & Lavoie, 2020). The screeners were more of a tool for risk assessments regarding the mental health crisis, not a proactive tool for policing or work safety. Instead, screeners were more connected to decision making on how to handle the situations; if the individual needed to be taken to health care, social services or be in custody (Hoffman et al., 2021; Sanders & Lavoie, 2020).

### Organizational factors

The implementation and use of these systems is also dependent on organizational factors, especially the management conditions. Sanders and Lavoie (2020) stress the importance of management’s involvement in the implementation, especially concerning ethical issues of such a system Also, practical matters, such as information sharing and documentation, need to be handled by involved organizations (Hirdes et al., 2020). Kane et al., (2018), in turn, raises the question of risks of flagging and over-flagging that needs to be handled within the organization. This is also addressed by Paterson et al. (2019). In addition, working with digital- or algorithm-based systems requires training, which often becomes an organizational assignment (Hoffman et al., 2016).

To implement and sustain digital and algorithm-based systems requires clear goals, motivation, technical expertise and properly trained professionals. In the three studies regarding health care-initiated system, workplace safety seemed to be an issue in contrast to the police-initiated system. For example, Paterson et al. (2019), shows the importance of streamlining the process and letting the health care professional be involved in the implementation. Hirdes et al. (2020) stresses that different actors in trans-institutional systems need to have different versions, to suit the specific mission and pre-conditions.

### Dilemmas and ethical issues

Dilemmas and ethical issues involved questions related to the individual’s integrity and the risk of stigmatization. Weinberger et al. (2018) highlights the risk of a shift in responsibility from health care to law enforcement, where flagging individuals as potential risk-patients may result in preventive measures that risk criminalization of emotional distress and displeasure, thereby replacing care with punitive responses. Haimovich et al. (2024) in turn, noted the risk of reinforcing bias and stigmatization of substance abuse, mental illness and ethnic minorities, resulting in decreased interactions and a less qualitative care.

In policing it is difficult to get informed consent about sharing information from the police systems to the emergency departments (Sanders & Lavoie, 2020). Kane et al. (2018) also observed that a mental health flag was a disadvantage, individuals with flags were significantly more likely to be charged and to spend a longer period in custody, compared to individuals without. The mentioned studies also addressed matters about who should have access to the system, which data should be used and for how long a flag would be active and, if digital- and algorithm-based alert system might reduce the will to seek help in emergencies (Kane et al., 2018; Paterson et al., 2019: Weinberger et al., 2018).

### Outlining the current knowledge

The use of digital- and algorithm-based alert systems seems to be used more in the health care sector compared to policing, particularly for addressing and preventing workplace safety issues (Haimovich et al., 2024). The use and implementation of systems in policing is motivated by improving police response (both interactions and efficacy), avoiding escalating encounters and sharing information from the police to the health care sector (Hirdes et al., 2020; Hoffman et al., 2016; Kane et al., 2018; Sanders & Lavoie, 2020). One study also pointed out that these systems can help justify budget allocations for non-crime events, as calls involving mental health consume significant resources, even though funding is often tied to crime statistics (Hoffman et al., 2021). Workplace safety for the police is not mentioned in any study as an argument or motive for implementation. One tentative explanation to this can be that the studies regarding the police has been conductive in a health care context, with one exception namely Sanders and Lavoie study from 2020.

On a general level the digital- and algorithm-based alert systems, which can either flag individuals or help to screen for mental illness, has a potential to improve the professional meeting approach, care and custody (Hirdes et al., 2020; Hoffman et al., 2016). It can also increase and improve communication within or between organizations involved, even though the direction of information often seems to flow from the police towards health care, rather than the other way around (Hoffman et al., 2016, Hoffman et al., 2021; Kane et al., 2018). In addition, this system may also help organizations to find a common language across professional boundaries, understand the different roles, bridge gaps between missions and prioritize the individual’s needs (Hirdes et al., 2020; Hoffman et al., 2016).

However, the use of these system needs to be conducted with caution, since there is also a risk for bias, stigmatization and “over-flagging” individuals (Haimovich et al., 2024; Kane et al., 2018; Paterson et al., 2019: Weinberger et al., 2018). This also shows the importance of organizational factors and understanding of these systems, namely the need for a clearly defined purpose, adequate training, involvement of the professional and organizational preparedness to handle both practical challenges and ethical considerations.

## Discussion

Considering the rapid technological development of digital and algorithm-based alert systems, this scoping review aimed to explore current knowledge about the use of such systems in health care settings and in policing, in situations involving individuals experiencing mental health crisis. The review included eight articles, which addressed systems for both policing and health care.

A central finding of the review is the differentiated rationale behind the implementation of these systems in health care compared to policing. The health care-initiated systems had a clearer purpose as they were often embedded within broader workplace safety strategies, but in policing the motivation for implementation were vaguer and more varied between studies. This is quite surprising, since both police- and health care professionals face similar challenges when interacting with individuals experiencing mental health issues and thus share a need to be psychologically ready for such encounters.

The systems described in the articles were also used in different ways; health care-initiated systems were mainly used for internal communication and to prepare staff for risk situations or potentially/possible violent encounters. In contrast, the police-initiated systems primarily consisted of various screening tools used during interactions to support decisions in the situation and to support collection of information for internal or external systems. The limited use of such systems as proactive safety measures for the police – unlike in health care – is a significant gap. This may reflect a cultural and institutional focus in policing on crime control rather than preventive care, which in turn may affect how such technologies are prioritized and integrated into practice. In addition, our result indicates that workplace safety in policing mental health crisis can be a neglected issue and need to be researched further.

The use of digital- and algorithm-based systems had both advantages and disadvantages and were also dependent on organizational factors to be functional and efficient. Leadership engagement, inter-agency trust, training, and clarity in protocols for data use were identified as essential. Several studies emphasized the importance of shared goals and mutual understanding between police and health care actors to enable meaningful collaboration. The use of alert systems in the welfare sector, especially policing to address mental health concerns, is a complex and multifaceted issue that requires careful consideration of ethical, technological, and legal implications. It introduces a range of ethical dilemmas and legal uncertainties. Concerns about integrity, consent, and the risk of stigmatization were raised in several of the studies. As discussed, the practice of “flagging” individuals as mentally ill or potentially dangerous may lead to reinforced biases, especially towards vulnerable groups such as individuals with substance use disorders, ethnic minorities, or socioeconomically marginalized groups. As shown in the reviewed articles, flagged individuals were disproportionately criminalized and often subjected to extended custody durations. These findings align with broader critiques of risk-based policing and the datafication of public services, where the use of predictive analytics has the potential to reproduce or even exacerbate systematic inequities.

Moreover, as highlighted earlier, the lack of transparency regarding how data is collected, categorized, shared, and retained poses serious concerns around legal accountability and public trust. Few of the reviewed studies examined whether individuals are even aware of being flagged or if they have any opportunity to contest or appeal such labels. This raises fundamental questions about due process, the right to be forgotten, and potential conflicts with existing legal data protection frameworks.

Despite these concerns, the review suggests that, when implemented thoughtfully – with clear protocols, training, and ethical oversight – digital and algorithm-based alert systems may support frontline decision-making, facilitate inter-professional understanding, and improve responses to mental health crises. As emphasized, the key lies not only in the technologies themselves, but in how they are designed, governed, and integrated into broader strategies. However, given the limited number of studies and their diverse context, these potential benefits require further empirical investigation.

Further research is needed to evaluate both the short- and long-term impacts of such systems on individual rights and institutional practices, and to develop frameworks that ensure their ethical, legal and equitable use. In addition, further research is needed to evaluate whether digital and algorithm-based alert systems may improve the work environment for the police, by allowing them access to more information in advance, thereby enabling them to be better prepared for an encounter.

### Limitations

Searches were limited to three databases and two additional search methods, and an inclusion of more databases may have identified additional studies for inclusion in the study. Due to the inclusion of a small number of studies in the analysis, the result and discussion are limited. However, despite the methodological limitations, the conducted scooping review contributes to the discussion on digital- and algorithm-based alert system by providing an over-view of the research field.

## Conclusion

This scoping review explored and synthesized current knowledge about the implementation and use of digital and algorithm-based alert systems in policing in relation to mental health crises. The review included both systems used by the police and health care-initiated systems used to alert the police.

The primary motivations for implementing such systems differed between police organizations and health care services. Within policing, motivations were mainly linked to information sharing between authorities, cost effectiveness, justification of budget for non-crime events, risk assessment and improving police response. In health care services, the main motivations were to enable both internal and external communication in risk situations and to directly enhance the safety of patient and staff.

Digital and algorithm-based alert systems have the potential to improve police responses and approach, facilitate better care and custody, and strengthen communication within and between organizations. Nevertheless, the use of these system needs to be conducted with caution, given the risks of bias, stigmatization, and “over-flagging” of individuals. Ultimately, our findings highlight the importance of carefully balancing the pursuit of efficiency with considerations of ethical implications and potential unintended consequences when implementing digital and algorithm-based alert systems. While it remains essential to respect individual integrity, it is also critically important to ensure that the police are properly equipped to respond to incidents where mental health concerns are present.

## Data Availability

No datasets were generated or analysed during the current study.

## Appendix 1

**Table:**

Article	Country	Method	Phenomena of interest
Haimovich, A.D., Taylor, R.A., Chang-Sing, E., Brashear, T., Cramer, L.D., Lopez, K., Wong, A.H. (2024).Disparities Associated With Electronic Behavioral Alerts for Safety and Violence Concerns in the Emergency Department. *Ann Emerg Med*. 83(2):100–107.	US	Quantitative	Health care initiated system
Hirdes, J.P., van Everdingen, C., Ferris, J., Franco-Martin, M., Fries, B.E., Heikkilä, J., Hirdes, A., Hoffman, R., James, M.L., Martin, L., Perlman, C.M., Rabinowitz, T., Stewart, S.L., Van Audenhove, C. (2020). The interRAI Suite of Mental Health Assessment Instruments: An Integrated System for the Continuum of Care. *Front Psychiatry.*17(10):article 926.	Canada	Quantitative	System for the police
Hoffman et al., (2021). Costs and Savings Associated With the Police Use of the interRAI Brief Mental Health Screener. *Front Psychiatry*.18(12):article 726,469.	Canada	Quantitative	System for the police
Hoffman, R., Hirdes, J., Brown, G.P., Dubin, J.A., Barbaree, H. (2016). The use of a brief mental health screener to enhance the ability of police officers to identify persons with serious mental disorders. *Int J Law Psychiatry*. 47:28–35.	Canada	Quantitative	System for the police
Kane, E., Evans, E., Mitsch, J., Jilani, T., Quinlan, P., Cattell, J., Khalifa, N. (2018). Police interactions and interventions with suspects flagged as experiencing mental health problems. *Crim Behav Ment Health*. 28(5):424–432.	England-Wales	Quantitative	System for the police
Paterson et al., (2019). Embedding Psychiatric Risk Flags Within an Electronic Health Record: Initial Findings and Lessons Learned. *Healthc Q.* 21(4):54–60.	Canada	Qualitative	Health care initiated system
Sanders, C. B., & Lavoie, J. (2020). Boundary objects and technological frames: officer’s’ perceptions and experiences using mental health screeners on the frontline. *Policing and Society.* 31(8):967–981.	Canada	Qualitative	System for the police
Weinberger, L. E., Sreenivasan, S., Smee, D. E., McGuire, J., & Garrick, T. (2018). Balancing safety against obstruction to health care access: An examination of behavioral flags in the VA health care system. *Journal of Threat Assessment and Management*. 5(1): 35–41.	US	Quantitative	Health care initiated system
